# Development and Evaluation of a Commercial Direct-Fed Microbial (Zymospore^®^) on the Fecal Microbiome and Growth Performance of Broiler Chickens under Experimental Challenge Conditions

**DOI:** 10.3390/ani12111436

**Published:** 2022-06-02

**Authors:** Otoniel F. de Souza, Bruno Vecchi, Emanuel Gumina, Fabrizio Matté, Fabio L. Gazoni, Xochitl Hernandez-Velasco, Jeffrey W. Hall, Catarina Stefanello, Sherry Layton

**Affiliations:** 1 Department of Animal Science, Federal University of Santa Maria, Av. Roraima, 1000, Santa Maria 97105-900, Brazil; otonielflx@hotmail.com (O.F.d.S.); catarina.stefanello@ufsm.br (C.S.); 2 Vetanco SA, Chile 33, Villa Martelli B1603, Argentina; bvecchi@vetanco.com (B.V.); egumina@vetanco.com (E.G.); sherry.layton@vetanco.com (S.L.); 3 Vetanco do Brasil Imp. e Exp. LTDA, Chapecó 89813-824, Brazil; fabrizio@vetanco.com.br (F.M.); gazonivet@yahoo.com.br (F.L.G.); 4 Departamento de Medicina y Zootecnia de Aves, Facultad de Medicina Veterinaria y Zootecnia, Universidad Nacional Autonoma de Mexico, Ciudad de Mexico 04510, Mexico; xochitlh@fmvz.unam.mx; 5 Vetanco USA, 1000 Westgate Drive, Ste 105, Saint Paul, MN 55114, USA; 6 BV Science, 8700 Monrovia, Suite 310, Lenexa, KS 66219, USA

**Keywords:** antibiotic growth promoter, broiler chicken, challenge conditions, direct-fed microbials, performance

## Abstract

**Simple Summary:**

Probiotics are recognized for their beneficial health-promoting properties, through competitive exclusion, promoting maintenance of intestinal epithelial integrity and host immune system homeostasis. The use of some spore-forming bacteria from the genus *Bacillus* has earned interest as a direct-fed microbial in recent years as a potential alternative to antibiotic growth promoters and growth enhancers. The present study evaluates the use of a *Bacillus subtilis* spore-based direct-fed microbial (Zymospore^®^, Vetanco, Villa Martelli, Argentina) compared to an antibiotic growth promoter on the performance of broiler chickens under experimental intestinal challenge conditions. The results suggest that Zymospore^®^ increases the diversity of the broiler fecal microbiota and is an acceptable substitute for commonly used antibiotic growth promoters under defined and non-defined intestinal dysbiosis conditions.

**Abstract:**

Direct-fed microbials (DFM) are added to broiler chicken diets in order to promote the proliferation of beneficial intestinal bacterial populations, which may lead to gains in performance efficiency and, potentially, reduce the level of enteric pathogens in the broiler chickens. The selection and laboratory evaluation of *Bacillus subtilis* strains as well as the experimental trial results of a novel *Bacillus*-based commercial DFM product are described. Fifteen wild-type *Bacillus subtilis* strains were characterized and assayed for their enzyme production capability, spore resistance to pH, salinity, and temperature, and ability to inhibit the growth of *E. coli* and *Salmonella* spp. The final DFM formulation was evaluated and compared to an antibiotic growth promoter (AGPs) in two experimental trials. In Experiment 1, broilers were given a defined challenge of *Eimeria* spp. and *Clostridium perfringens* to induce intestinal dysbiosis. The optimal dose of the DFM was determined to be 0.3 kg/ton of feed. At this dose, the broilers fed the DFM performed as well as the Flavomycin^®^-fed broilers. Further, intestinal microbiome analysis indicates that the use of the DFM enhances bacterial diversity of the gut flora by day 5 of age, increasing levels of lactic acid bacteria (LAB) and Clostridiales by 25 days of age, which may enhance the digestion of feed and promote growth of the birds. In Experiment 2, the broilers were raised on recycled litter and given an undefined challenge orally to mimic commercial growth conditions. In this trial, the DFM performed as well as the bacitracin methylene disalicylate (BMD)-11%-fed birds. The results of the present studies suggest that this novel DFM, Zymospore^®^, improves the performance of broiler chickens under experimental challenge conditions as effective as an AGP, providing a safe and effective substitute to the poultry industry.

## 1. Introduction

Intensive management practices in poultry production induce enteric microflora imbalances leading to diminishment of performance parameters [1,2]. To alleviate the effect of dysbiosis in the gastrointestinal tract, diets have been commonly supplemented with antibiotic growth promoters (AGPs), demonstrating an effective decrease in the presentation of digestive disorders [3], though the underlying mechanism of this AGP-driven enhancement is not well understood. Recent microbial genomics and metabolomic analysis of the broiler cecum indicates AGPs alter the bacterial community of the ceca, increasing the overall microbial gene content of the cecum, which enhances the bacterial community’s ability to recycle host nitrogen compounds [4]. Further, it appears that AGP-modified bacterial communities promote increased levels of bile salt production, helping the host absorb fatty acids [4]. Both processes, in turn, drive performance of the broilers. Identification of novel non-antibiotic compounds and/or mixtures that have the same positive impact on performance is of great importance to the broiler as the concern for antimicrobial resistance (AMR) grows and bans on the use of AGPs spread around the world [5].

The indiscriminate and inappropriate use of antibiotics has led to the emergence of multidrug-resistant pathogens, resulting in a ban on many AGPs [6,7]. As an alternative to AGPs, probiotics have been under investigation as feed additives to modulate the intestinal microflora, which in turn support good productive responses in animals [8,9]. Among the species of microorganisms used as probiotics, some strains of the facultative anaerobic Gram-positive genus *Bacillus* are receiving important attention due to their augmentative properties on digestion, absorption of nutrients, and intestinal morphology [4,10,11]. Furthermore, the control of enteropathogens such as *Salmonella* spp., *Clostridium perfringens*, *Campylobacter* spp., and *Escherichia coli* in the gastrointestinal tract (GIT) has been associated with the use of *Bacillus*-based probiotics [12,13]. The genus *Bacillus* has the extraordinary capacity to produce endospores under stressful environmental conditions; some of these spores can resist high temperatures used during feed preparation (pelletization), extreme pH, dehydration, high pressures, and contact with caustic chemical substances [14]. These admirable features make selected *Bacillus* spores a direct-fed microbial (DFM) suitable for commercialization and distribution due to their AGP-like performance improvements, long shelf-life, and stability [15,16].

There is evidence supporting the theory that some *Bacillus* spores germinate in the GIT of chickens [17], mice [18], pigs, dogs, and humans [19]. Metabolically active cells are believed to produce antimicrobial substances, have immunomodulatory effects on the intestinal mucosa, and function as competitive exclusion agents interacting with host cells [20]. Furthermore, some *Bacillus* species can produce and export an array of extracellular enzymes, including protease, phytase, xylanase, keratinase, lipase, and cellulase [21,22]. These enzymes help to degrade complex feed molecules, improve absorption of nutrients, reduce intestinal viscosity in non-starch polysaccharide-rich diets (NSP), and decrease the amount of substrates available for the growth of pathogenic bacteria [23,24,25]. Additionally, it has been shown that the presence of *Bacillus* species, such as *Bacillus subtilis*, enhances the growth of other beneficial microorganisms, for example, *Lactobacillus*, by the production of subtilisin and catalase and also by decreasing intestinal pH [26].

All the benefits related to the utilization of *Bacillus*-DFMs in the diet make supplementation with *Bacillus* spores an accessible and applicable alternative to antibiotic growth promoters, while avoiding a concomitant increase in gastrointestinal diseases and maintaining or improving performance parameters in poultry production under commercial conditions. The purpose of the present study was to evaluate a recently developed commercial direct-fed microbial (Zymospore^®^) relative to AGPs on the performance of broiler chickens under experimental intestinal challenge conditions. Further, the fecal microbiome of a subset of birds was evaluated by 16S DNA sequencing. The data revealed that the DFM-fed birds had a greater abundance and diversity of bacteria in their feces than the basal-diet-fed birds, a feature similar to AGP-fed birds [4]. The growth performance of broilers fed the DFM in these studies was better than the basal-diet-fed controls and similar to the performance of the AGP-fed birds. 

## 2. Materials and Methods

### 2.1. Characterization of Bacillus subtilis Strains 

Fifteen strains of *Bacillus* spp. previously isolated from soils collected from around the country of Argentina were initially screened on tryptic soy agar (TSA, Britania Labs, Caba, Argentina) and Spizizen potato agar (SPA, ATCC medium 423) plates for their ability to inhibit the growth of *Salmonella enterica* serovar Enteritidis (*S*. Enteritidis, SE), *S.* Typhimurium (ST), *Escherichia coli* isolated from pigs afflicted with colibacillosis (EC-P), an avian pathogenic *E. coli* (APEC) isolate (EC-C), and an enteropathogenic *E. coli* (EPEC) isolate (EC-E) as previously described in [24] with the simple modification of using a sterile 1 µL loop to streak a straight line of each *Bacillus* isolate onto individual agar plates that did not touch the edge of the dish (2 agar plates per *Bacillus* isolate, 5 pathogen isolates). Each plate was overlaid the following day with the respective pathogen isolate of interest and incubated for 24 h at 37 °C. Zones of inhibition were measured and scored as follows: (−) no inhibition, (+) inhibition < 2.0 cm, (++) inhibition 2.1 to 4.5 cm, (+++) inhibition > 4.6 cm from edge of *Bacillus*, respectively. As growth and inhibition were best on SPA plates, *Bacillus* isolates were evaluated for each pathogen inhibition on SPA agar (Supplementary Table S1). An example (Figure S1) is provided in the Supplementary Data. Further, the *Bacillus* isolates (Table 1) that consistently ranked among the best for pathogen growth inhibition were selected for further characterization and were evaluated using enzyme-specific agar plates to detect extracellular enzyme production [24]. The spores of the strains were assayed for their resistance to temperature, salinity, and pH [14]. Based on a combination of pathogen growth inhibition and enzyme production, three strains were selected and the colony morphology and growth characteristics of the three strains on TSA plates were observed and recorded. Each strain was positively identified as *Bacillus subtilis* using a series of macroscopic, microscopic, and biochemical assays (bioMerieux API 50 CHB test kit, and bioMerieux, Lyon, France) (data not shown). 

Evaluated strains were assayed for the following extracellular enzymes: amylase (starch agar, Difco™ Starch Agar, BD, Franklin Lakes, NJ, USA), cellulase (carboxymethyl cellulose agar, ATCC medium: 1513), proteases (casein agar, Remel™, Lenexa, KS, USA) [27], and lipases (Difco™ Spirit Blue Agar, BD, Franklin Lakes, NJ, USA). The strains were cultured in tryptic soy broth (TSB, Britania Labs, Caba, Argentina) overnight at 37 °C, centrifuged and washed with 0.9% sterile saline the following day. The culture was quantified by serial dilution on TSA plates and stored at 4 °C overnight. The following day, each culture was diluted to 10^8^ CFU/mL with sterile saline. Subsequently, 10 µL of each strain was placed at the center of each respective enzyme assay agar plate, allowed to absorb, and incubated at 37 °C overnight. To detect amylase and cellulase enzyme activity, the plates were flooded with Lugol solution (Sigma-Aldrich, St. Louis, MO, USA) for 2 min and 1 M NaCl (Sigma-Aldrich, St. Louis, MO, USA) solution for 15 min, respectively. Excess solution was discarded, revealing potential zones of clearing in the agar indicative of the respective enzyme activity. A relative enzyme activity (REA) score was used to categorize the strains into Excellent, (REA > 5); Good, (REA 2 < 5); and Poor, (REA < 2) enzyme producers. The REA is calculated by dividing the diameter of the zone of clearing by the diameter of the colony in cm (REA = Ø of the zone of clearing (cm)/Ø colony (cm)) [24]. Each strain was analyzed by triplicate plating.

Spores of the selected *Bacillus subtilis* strains were assayed for their ability to withstand potential physiological environments found within birds for two and four hours. An isolated colony of each strain was inoculated into Difco Sporulation Medium (DSM, BD, Heidelberg, Germany) and incubated at 37 °C for 3 days. Sporulation was confirmed by microscopy and malachite green (Sigma-Aldrich, St. Louis, MO, USA) staining. The vegetive colony-forming unit capability of each spore preparation was determined using serial dilution on TSA plates. Subsequently, each spore preparation was diluted to 10^8^ CFU/mL. Subsequently, 1 mL of the spore solution was added to 9 mL of sterile saline adjusted to the indicated salinity (NaCl, Sigma-Aldrich, St. Louis, MO, USA) or pH (1 M HCl, Sigma-Aldrich, St. Louis, MO, USA) in sterile borosilicate glass tubes and incubated at 37 °C. For the temperature resistance analysis, physiological saline was used. At 2 and 4 h of incubation, the tubes were vortexed, and 0.2 mL of the solution was serially diluted on TSA to determine the vegetative CFU/mL as an indirect measure of spore resistance to each treatment condition. These analyses were performed in triplicate. 

### 2.2. Direct-Fed Microbial (DFM) Product

Zymospore^®^ (Vetanco S.A., Buenos Aires, Argentina) is a *Bacillus subtilis* spore-based direct-fed microbial (DFM) containing at total of 5 × 10^11^ spores/gram from BS-009, BS-020, and BS-024.

### 2.3. Experiment 1

#### 2.3.1. Housing Conditions 

Experiment 1 was conducted at the Federal University of Santa Maria, Santa Maria, Brazil. Birds were reared in floor pens (1.5 m^2^) with new wood shavings as litter in a clean experimental university poultry house. Each pen was equipped with one 18 kg feeder and one drinker. The average temperature was 32 °C at placement, being reduced by 1 °C every two days until reaching 23 °C to provide comfort throughout the study. A continuous lighting schedule was used until d 7 post-hatch, whereas an 18L: 6D cycle with constant intensity was used after that. Birds had ad libitum access to water and mash feeds. All procedures used in the current study were approved by the Federal University of Santa Maria, Santa Maria, Brazil (number 5404280717). 

#### 2.3.2. Animal Source and Diets

Seven hundred and fifty slow feathering male broiler day-old chicks (Cobb 500), vaccinated for Marek’s and Avian Bronchitis diseases at the hatchery, were purchased from a local hatchery (Agrodanieli Group, Tapejara, RS, Brazil) and weighed (45 g ± 0.4 g). A four-phase corn–soybean-meal-based feeding program was used with pre-starter (1 to 7 d), starter (7 to 21 d), grower (21 to 35 d), and finisher (35 to 42 d) diets formulated according to Rostagno et al. [28] (Table 2) with or without additives. All chickens were weighed on days 1 and 42 to calculate body weight gain (BWG). Pen feed intake (FI) from days 1 to 42 was recorded to calculate the feed conversion ratio (FCR, d 42 Pen BW/accumulated (d1–42) Pen FI) at the end of the trial. Mortality was recorded daily.

#### 2.3.3. Experimental Design

Broiler chickens were distributed into 5 experimental groups with 6 replicates of 25 birds in a completely randomized design as shown below. Group 1 (basal diet, negative control) received commercial diets with no additives and no challenge. Group 2 (0.2 kg/ton) received the basal diet with the addition of 0.2 kg of DFM per metric ton of feed, Group 3 (0.3 kg/ton) received the basal diet with the addition of 0.3 kg of DFM per metric ton of feed, Group 4 (0.4 kg/ton) received the basal diet with the addition of 0.4 kg of DFM per metric ton of feed, and Group 5 (AGP, positive control) received the basal diet with the addition of 0.025 kg of flavomycin per metric ton of feed. Groups that received the DFM or AGP received it in all feed phases. All chickens were weighed on days 1 and 42 to calculate BWG. To calculate the FCR at the end of the trial, the Pen BW (d 42)/accumulated Pen FI (d1–d42) was utilized. Mortality was recorded daily.

#### 2.3.4. Challenge Model to Induce Dysbiosis

All broilers were challenged on day 14 via individual oral gavage with 10× the regular dose of a commercially approved coccidian vaccine (Bio-Coccivet R^®^ live vaccine, containing *Eimeria acervulina*, *E. brunetti*, *E. maxima*, *E. necatrix*, *E. praecox*, *E. tenella*, and *E. mitis*; Biovet Vaxxinova, Vargem Grande Paulista, Brazil). At 19 days, all birds were individually orally gavaged with 1 mL/bird of *Clostridium perfringens* toxin Type A at an analyzed concentration of 3.1 × 10^9^ CFU/mL (UFPR, Curitiba, Brazil). This intestinal challenge model has been described to induce intestinal dysbiosis [29,30]. To obtain the *Clostridium perfringens* inoculum, 50 µL of the isolate from the bacterial stock was statically cultured in 3 mL of tryptic soy broth (TSB) (BD, Heidelberg, Germany) in a BD GasPak jar equipped with GasPak H2 + CO_2_ generator envelopes and catalyst at 37 °C for 24 h. Then, the isolate was streaked across ten tryptic soy agar (TSA) plates (BD, Heidelberg, Germany) and cultured in a GasPak Jar as described above for 24 h in an incubator at 37 °C. The bacterial lawns were sterilely collected with cell scrapers and added to 25 mL of ice-cold sterile PBS. The inoculum was vortexed and centrifuged at 5400× *g* for 20 min at 4 °C. The cells were washed once and diluted into 800 mL of PBS and kept on ice until use. The CFU of the inoculum was determined by serial dilutions on TSA plates and cultured as described above.

#### 2.3.5. Sampling, DNA Extraction, Sequencing, and Bioinformatic Analysis

Fecal samples from the three different treatments (negative control, NC; 0.2 kg of DFM/ton of feed and 0.4 kg of DFM/ton of feed) at two different sampling points (5 and 25 days of life) were taken, and in total, 36 samples were analyzed. Each sample contained approximately 50 g of pooled, fresh feces from each replicate for each treatment. The d5 and d25 time points were chosen to evaluate potential changes in the fecal microbiome at an early and mid-life stage of the broilers at the minimum and maximum concentration of the DFM used in the experiment. 

Fecal samples were processed (Imunova, Curitiba, Brazil) with the ZR Fecal DNA Miniprep kit (Zymo Research, Tustin, CA, USA) following the manufacturer’s instructions. The extracted DNA was quantified by spectrophotometry at 260 nm with a NanoDrop^®^ 2000 (ThermoScientific, Wilmington, DE, USA) spectrophotometer. To verify the integrity of the DNA, all the samples were evaluated with an agarose gel electrophoresis, stained with ethidium bromide (1% *w*/*v*, Glentham Life Sciences, Corsham, UK), and visualized with UV light. 

A 250 base pair segment of the hypervariable V4 region of the ribosomal 16S rRNA gene was amplified using universal primers 515F and 806R with the following PCR conditions: 94 °C for 3 min; 18 cycles of 94 °C for 45 s, 50 °C for 30 s, and 68 °C for 60 s; followed by 72 °C for 10 min. From these, a metagenomic library was constructed using the commercial Nextera DNA Library Preparation Kit (Illumina, Hayward, CA, USA). The amplicons were pooled and sequenced using the Illumina MiSeq sequencing system [31]. To facilitate data visualization, the second sampling time (d25) was tagged as “b”.

Sequencing data files were transferred, and analysis was conducted by the University of Minnesota Genomics Center. Sequence files were de-multiplexed with BBMap (https://sourceforge.net/projects/bbmap/; demuxbyname.sh, accessed on: 8 September 2021) and further processed in DADA2 (https://benjjneb.github.io/dada2/tutorial.html, accessed: 8 September 2021). The filter and trim parameters were as follows: maxN = 0, maxEE = 2, truncQ = 2, rm.phix = TRUE. The DADA2 algorithm was run with pseudo-pooling and chimeras were removed with the consensus method in ‘remove BimeraDenovo’ before assigning taxonomy using DADA2 ‘assignTaxonomy’ and ‘addSpecies’ using the maintained databases of ‘rdp_train_set_18.fa.gz’ and ‘rdp_species_assignment_18.fa.gz’, respectively. 

Further data analysis was performed in R. The beta diversity analysis used a customized CLR transform (https://github.com/trevorjgould/dada2_pipeline.git, accessed on: 8 September 2021) followed by PCA. The alpha diversity Simpson and Shannon indexes used the ‘vegan package’ (https://cran.r-project.org/web/packages/vegan/vegan.pdf, accessed on: 8 September 2021). The Chao1 index used the ‘OTUtable’ package. All plots used ggplot2, reshape2 (https://github.com/hadley/reshape, accessed on: 8 September 2021), and dlpyr (https://dplyr.tidyverse.org, accessed on: 8 September 2021) for processing. 

For statistical analysis, samples were CLR transformed, and Analysis of Similarity (ANOSIM) was performed on Aitchinson distance. Indicator species analysis was performed using the multipatt function in the ‘indicspecies’ R library with 9999 permutations while controlling for multiple test corrections using Benjamini–Hochberg correction. The ‘adonis’ function was performed on CLR transformed data with Euclidean distance. Betadisper function was tested and pairwise adonis function was performed on the three treatment groups for d5 and d25, separately, with multiple test correction using Benjamini–Hochberg formula. Data visualization utilized R and GraphPad Prism 9. 

### 2.4. Experiment 2

#### 2.4.1. Housing Conditions 

This experiment was conducted at the experimental farm of Bioinnovo in Buenos Aires, Argentina. The broiler barn is an open-sided 600 square meter facility with a concrete floor housing 48 pens divided into 3 lines of 16 pens each. Each pen is 2.5 m^2^ and equipped with individual feeders, individual in-line medicators, and fresh water. The heat is provided via air heaters, and the facility has six fans for heat relief. Each pen contained wood shavings 15 cm high, composed of 50% new wood shavings and 50% reused from previous experiments. The density, lighting program, and temperature were maintained within optimal parameters as outlined (www.cobb-vantress.com, accessed on 19 August 2019). Animal care was provided by an on-staff veterinarian. 

#### 2.4.2. Animal Source and Diets

Four hundred one-day-old male broiler chickens (Cobb 500) were obtained from a commercial hatchery and were vaccinated for Newcastle Disease and Marek’s Disease at the hatchery. All birds received feed and water ad libitum. The study included two commercial diets in the form of micropellets, pre-starter from days 1 to 14, and finisher from day 15 to 42, the end of the trial (Table 3). Husbandry conditions such as environmental temperature and the light program were adjusted to the recommended guidelines of the genetic line. All animal handling procedures followed the guidelines of the Institutional Committee of use and care of experimental animals of the National Institute of Agronomic Technologies (INTA), protocol number 6/2021.

#### 2.4.3. Experimental Design

In this experiment each treatment group contained 100 birds per group, divided into 5 repetitions with 20 birds/repetition. Group 1 (basal diet) received commercial diets with no additives and no challenge. Group 2 (BMD−) received commercial diets with the addition of 0.5 kg/metric ton of bacitracin methylene disalicylate (BMD) 11% in all the feed phases and no challenge. Group 3 (BMD+) received commercial diets with the addition of 0.5 kg/metric ton of BMD 11% in all the feed phases and was challenged with the litter filtrate. Group 4 (DFM+) received commercial diets with the addition of the DFM and Zymospore^®^ at an inclusion rate of 0.3 kg/metric ton in all the feed phases and was challenged with the litter filtrate. All chickens were weighed on days 1 and 42 to calculate BWG. To calculate the FCR at the end of the trial, the Pen BW (d 42)/accumulated Pen FI (d1–d42) was utilized. Mortality was recorded daily.

#### 2.4.4. Litter Filtrate to Recapitulate Commercial Farm Conditions 

On days 7, 21, and 22 of life, chickens in specified groups received in the drinking water a liter filtrate as described by Sakomura and Rostagno [32]. Ten kilograms of reused litter (2 cycles minimum) from a commercial farm with a high historical prevalence of necrotic enteritis (75,000 oocysts per gram of litter) was mixed into fifty liters of distilled water at 22 °C for twenty-four hours. The solution was filtered through a stainless-steel metallic mesh with holes of 0.5 mm in diameter. The filtered solution was left untouched for one hour. One liter of this solution was then diluted with four liters of distilled water and administrated in the drinking water to each pen during a five-hour period on days 7, 21, and 22 of age. This procedure was repeated for each day of filtrate administration. The final dilution’s average microbial count revealed that a liter of filtrate contained 3 × 10^9^ CFU of total aerobic bacteria; 2 × 10^7^ CFU of total anaerobic bacteria; and 7 × 10^6^ CFU of total coliforms.

### 2.5. Statistical Analysis

All performance data were subjected to analysis of variance (ANOVA) as a completely randomized design using the general linear model (GLM) procedure of SAS [33]. For evaluation of growth performance parameters (body weight (BW), BWG, FI, and FCR), each of the replicate pens were considered as the experimental unit in each experiment, respectively. Treatment means were partitioned using Tukey’s multiple range test with an alpha threshold set at ≤0.05, indicating statistical significance. Experiment 1: broiler chickens were distributed into 5 experimental groups with 6 replicates of 25 birds in a completely randomized design. Experiment 2: broiler chickens were distributed into 4 experimental groups with 5 replicates of 20 birds in a completely randomized design.

## 3. Results

### 3.1. Bacillus Species Characterization and Stain Selection

A selection of *B. subtilis* isolates were evaluated for their ability to inhibit the growth of pathogenic *Salmonella* and *Escherichia coli* isolates on TSA and SPA plates as an indirect measure of their potential probiotic effect on broiler chickens and other animals (Supplementary Figure S1 and Table S1). Ultimately, three growth compatible strains, BS-009, BS-020, and BS-024 (data not shown) were selected for inclusion into the direct-fed microbial (DFM). Their colony morphology on solid agar media (Table 1) and vegetative cellular and spore characteristics and were observed microscopically (data not shown). All three strains presented as Gram-positive bacilli consistent with *B. subtilis*, which formed endospores as determined by malachite green staining (data not shown). Biochemical and fermentative characteristics of each strain were consistent with *B. subtilis* (data not shown). Colony morphology was similar among the three isolates, each exhibiting rough yellow-white colonies with irregular and lobate edges (Table 1). All three strains secreted various amounts of cellulase, amylase, lipase(s), and proteases (Table 4). The spores of the three strains exhibited resistance to temperature (Table 5), salinity (Table 6), and acid (Table 7). The spores of the three strains were highly resistant to all three stressors after two and four hours with each strain maintaining a vegetative CFU count consistent with the original spore inoculation of 10^7^ spores/mL.

### 3.2. Experiment 1

#### 3.2.1. Necrotic Enteritis Challenge and Performance Data

In addition to the food safety pathogens *Salmonella* and *E. coli*, broilers commonly face diseases such as coccidiosis and necrotic enteritis (NE) that cause intestinal dysbiosis, leading to reduced growth performance and increased production costs for farmers [34]. To evaluate potential disease mitigating effects of this newly developed DFM on broilers in the face of a performance reducing disease, a controlled NE challenge model was used in a pilot study to induce intestinal dysbiosis. While severe outbreaks of NE may cause up to 50% mortality in a flock, sub-clinical NE leads to diarrhea, dehydration, decreased feed consumption, and overall poorer performance of the broilers [35]. Further, the fecal microbiome of the broilers from a subset of the treatments was analyzed to evaluate changes in the bacterial community. 

Table 8 shows the results of this pilot study, Experiment 1. There is a lack of statistical significance among any of the groups across the different parameters. Of note, throughout the study, particularly after the NE challenge was applied, the DFM and AGP groups had numerically better parameters than the basal diet. Particularly after day 21 of life, the NC + 0.3 kg DFM/metric ton of feed group had similar or better numerical total BW and BWG parameters compared to the AGP group, with both groups numerically better than the basal diet group. Further, the accumulated FCR and FI of the NC + 0.3 kg DFM/metric ton of feed group were numerically as good or better than the AGP group. Interestingly, the NC + 0.2 kg DFM/metric ton of feed and NC + 0.4 kg DFM/metric ton of feed groups numerically improved parameters over the basal diet, but the data suggest there is an optimal dosage of 0.3 kg Zymospore^®^/metric ton of feed. In this experiment, no significant differences were observed in mortality.

#### 3.2.2. Fecal Microbiome Analysis

The fecal microbiome of a limited set of treatment groups was analyzed to help understand potential intestinal microbiome changes that may be occurring due to administration of the DFM. The groups analyzed were the basal diet (NC), NC + 0.2 kg/metric ton, and NC + 0.4 kg/metric ton groups. Fresh feces from each replicate within each treatment group was collected and pooled on days 5 and 25 of life. The samples were analyzed by 16S rDNA sequencing to evaluate bacterial changes that may be occurring within the broiler gastrointestinal tract.

**Figure 1:**
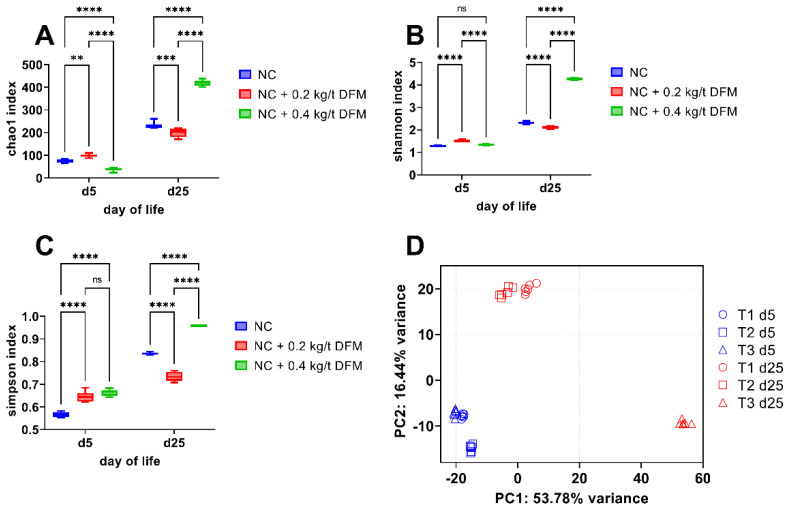
Alpha and beta diversity analysis. Levels of alpha diversity calculated as (**A**) Chao1, (**B**) Shannon, and (**C**) Simpson indices are depicted as boxplots by treatment group; d 5 and d 25 refer to day of life. (**D**) Bray-Curtis beta diversity shown as a principal component analysis (PCA). ** *p* < 0.01, *** *p* < 0.001, **** *p* < 0.0001.

**Figure 2:**
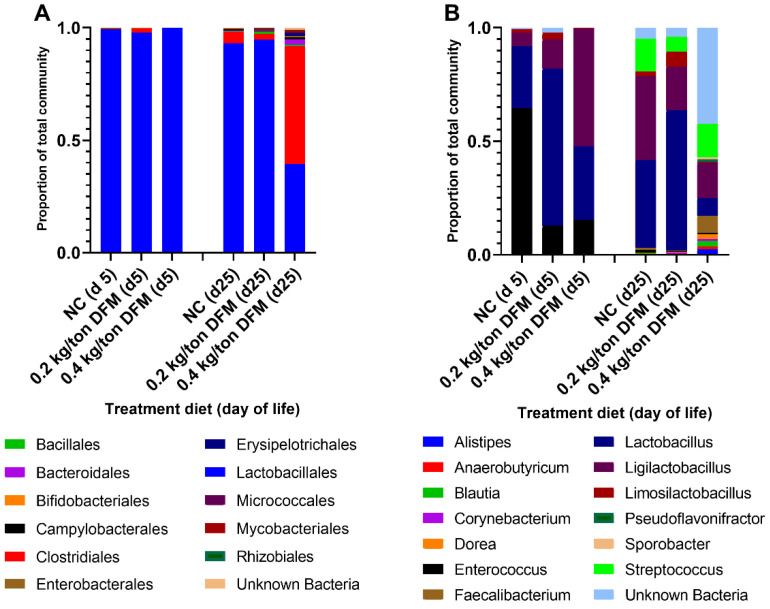
Biodiversity and proportionality of bacteria among treatment groups at d 5 and d 25 of life in broilers. Relative abundances of bacterial (**A**) Orders and (**B**) Genus among treatments within sampling days. When an ASV could not be resolved to a single taxon it was marked “Unknown” and resolved to the next highest taxa level. Unresolved taxa were grouped into “Unknown Bacteria”. For genus-level data, only genera with ≥1% abundance are shown.

The fecal bacterial community of all treatment groups was dominated by a few genera on d 5 (prior to challenge) of life with diversity increasing by varying degrees at d 25 (post-challenge) of life (Figure 1A–C). The level of alpha diversity as visualized by the Chao1, Shannon, and Simpson indices (Figure 1A–C), while statistically different, was relatively similar at d 5 in all treatment groups and was dominated by three genera, *Enterococcus*, *Lactobacillus*, and *Ligilactobacillus*. By d 25, the richness of taxa within each treatment group increased, as shown by the alpha diversity indices (Figure 1A–C). It is notable across all the indices at d 25, that the NC + 0.4 kg/metric ton DFM group results are roughly twice that of the NC and NC + 0.2 kg/metric ton groups, indicating greater species richness, but many of these genera are in low abundance (Figure 1A–C and Figure 2A,B). Beta diversity analysis, displayed as a principal component analysis (PCA), indicates the d 5 samples cluster together, but the dominance of *Enterococcus* in NC + 0.2 kg/metric ton is highlighted by a small divergence from NC and NC + 0.4 kg/metric ton groups (Figure 1D). In the PCA, the NC + 0.4 kg/metric ton is significantly dissimilar to the NC and NC + 0.2 kg/metric ton groups, which group together, indicative of greater taxonomic diversity (Figure 1D).

Taken together, the alpha and beta diversity indices indicate the DFM minimally impacted the diversity of the fecal microbiome early in life and prior to the necrotic enteritis challenge. The NC + 0.4 kg/metric ton group has greater species richness and diversity as compared to NC and NC + 0.2 kg/metric ton groups, with the treatment group explaining 57.78% of the variance between groups. The microbiota of NC and NC + 0.2 kg/metric ton groups were dominated by the Order Lactobacillale (Figure 2A,B), with *Lactobacillus* and *Enterococcus* contributing >80% of the total amplicon sequence variants (ASV) detected. To a lesser extent, the NC + 0.4 kg/metric ton also had a high proportion of Lactobacillale at d 5, but the distribution includes additional Lactobacillaceae, specifically *Ligilactobacillus* (Figure 2B), and to a lesser extent, *Enterococcus* compared to NC and NC + 0.2 kg/metric ton groups (Figure 2B). At d 25, the diversity of the NC + 0.4 kg/metric ton microbiota is underscored by the increased proportions of additional taxa as compared to NC and NC + 0.2 kg/metric ton groups (Figure 2B and Figure S2). This greater diversity comes as the expense of *Lactobacillus* and *Enterococcus* genera, which make up a significantly smaller proportion of the microbiota in the NC + 0.4 kg/metric ton group by d 25 (Figure 2B and Figure S2) but includes an increase in the proportion of other Families of the Order Lactobacillales (lactic acid bacteria) and Clostridiales, such as Faecalibacterium (Figure 2B and Figure S2).

The results of Experiment 2 comparing an AGP (11% BMD) to the DFM on BW, FI, FCR, and total mortality in broiler chickens given a non-defined litter filtrate are summarized in Table 9. In this experiment, the use of (−) and (+) after the group acronym is to indicate if a specific group did not or did receive the litter filtrate via drinking water, respectively. The basal diet (NC−) group did not receive the litter filtrate or feed additive and established the basal growth performance parameters of the trial. The BMD-positive control group not receiving the litter filtrate (BMD−) showed a significant improvement (*p* < 0.05) in BW and FCR when compared to the NC− group, as expected. Notably, the BMD+ and DFM+ groups, which received the litter filtrate, were highly similar to each other across BW, FI, and FCR parameters and were statistically better than the NC− group even though BMD+ and DFM+ received the undefined litter challenge. No mortality differences were observed among the groups.

### 3.3. Experiment 2

#### Intestinal Dysbiosis Challenge and Performance

It is a common practice to reuse litter on commercial farms, whereby fresh litter is laid over the top of used litter. This practice, while cost saving, perpetuates the cycling of potential pathogenic microorganisms through the new broiler flocks, thus the growth performance of new chicks may be impacted by sub-clinical diseases. To recapitulate this diverse and sub-clinical challenge of a commercial farm setting, an undefined litter filtrate was given via the drinking water in Experiment 2 (see Methods) to establish the cycling of microorganisms in the chicks’ excreta and litter. Based on results from Experiment 1, a single concentration of the DFM at 0.3 kg/t of feed was used. 

## 4. Discussion

The *Bacillus* genus is a phenotypically and genetically diverse endospore-forming taxa found within many ecosystems. Species of *Bacillus* produce a plethora of exo-proteins and -enzymes as well as bacterial antagonistic factors, including antibiotics [36,37]. The resistance of spores to environmental factors, cleaning agents, and sterilization methods as well as their diverse physiological properties make *Bacillus* of great interest to the food, animal, and biotechnology sectors. Specifically, the use of some spore-forming bacteria from the genus *Bacillus* have earned interest as direct-fed microbials in recent years as potential alternatives to AGPs. Inclusion of *Bacillus*-DFMs in broiler diets has been shown to have positive effects on the overall performance of boilers, the broiler immune system, and their resistance to disease [23,25,34,38,39,40,41]. The resilient capacity of spores to resist harsh environmental conditions, as well as their long shelf-life, make them feed-stable and suitable for commercialization in human and animal health and nutrition [42,43]. 

Nevertheless, it is essential to understand that not all *Bacillus* species can be used as DFMs. Each isolate has unique genetic and phenotypic characteristics, which in turn influence changes in the intestinal tract and the isolates’ heat resistance capacity, rate of growth, sporulation rate, and persistence in the GIT [18,44]. Herein, we described the characterization and development of a novel *Bacillus*-based DFM that increased the diversity of the broiler fecal microbiota and performed as well or better than commonly used AGPs under defined and non-defined intestinal dysbiosis conditions. 

A common feature of *Bacillus* species is their ability to inhibit the growth of other bacteria to varying degrees. Of interest to the poultry industry is the inhibition of colonization or growth of food-borne pathogens such as *Escherichia coli* and *Salmonella* species, among others, in or on the broiler. The novel strains isolated in this study, to varying degrees, displayed the ability to inhibit the growth of these pathogens in vitro (Figure S1 and Table S1), consistent with other *Bacillus* species isolated and characterized previously [45,46]. This antagonistic effect on the growth of other bacteria is associated with the production of a variety of natural antibiotics [36,47].

*Bacillus subtilis* produces a wide array of secreted enzymes known to promote and optimize the digestibility of non-starch polysaccharide (NSP)-rich diets such as xylanases, cellulases, and β-glucanases [23,24,48]. The inclusion of specific *Bacillus*-DFM candidates that produce exogenous enzymes, such as cellulases, amylases, and xylanase, in high NSP diets significantly reduced both viscosity and *C. perfringens* proliferation in an in vitro digestive model study simulating different compartments of the GIT [49]. The selected strains for this novel DFM produce extracellular enzymes, proteases, and lipases (Table 4) and were highly resistant to simulated GIT conditions (Table 5, Table 6 and Table 7) which may aid in transiting the GIT and, upon sporulation, facilitate the degradation of low-quality proteins and fats present in the diet that are used by the host for growth and prevention of detrimental enteric microflora changes. It was observed that the strains evaluated in this work had varying levels of enzyme production. Direct empirical comparisons are difficult because of the relative assay measurements, but recent work analyzing the relative enzyme activity of *B. subtilis* isolated from the broiler chick GIT identified a wide range of enzyme production capabilities in the isolates [45]. This is not unsurprising, as the regulation and secretion of exoproteins in Bacilli is multi-factorial and complex.

The use of *Bacillus* species probiotics and DFMs as alternatives to AGPs to enhance growth performance metrics is well established. We tested this new *Bacillus* DFM formulation for broiler growth enhancement in two separate studies using different methods (1, necrotic enteritis model; 2, reused litter and oral challenge model) to disrupt intestinal homeostasis and stunt the growth of the chicks. 

In Experiment 1, a previously defined NE challenge model [29,30] was utilized to examine the in vivo effects of this new DFM on performance parameters. Chickens supplemented with the *Bacillus*-DFM had similar growth performance parameters as those chickens supplemented with the AGP (flavomycin), which were greater than the basal control diets (Table 8). The lack of statistically significant differences in this trial is directly related to the limited replication and number of birds used in the trial. On an individual bird level, marginal gains in performance may seem nominal but accumulated across a large number of birds, and the impact can produce significant cost savings and economic returns to producers in the form of greater raw meat production and reduced feed costs. Based on these data of Experiment 1, the optimal dose of this new DFM formulation was determined to be 0.3 kg of DFM per metric ton (0.3 kg/ton). 

In Experiment 1, a resource-limited number of samples were analyzed to survey potential impacts of the DFM on the fecal microbiome consortium of the chickens at the minimum and maximum doses of the trial. Inclusion of the DFM into broiler diets altered the fecal microbiome of broilers as early as five days of age. At an inclusion rate of 0.4 kg/ton, the DFM suppressed the early dominance of *Lactobacillus* and *Enterococcus* and promoted greater diversity in species abundance by day 25. Within the greater context of the effect of *Bacillus* DFMs on the fecal microbiome and the relationship to body weight gain and feed conversion efficiency, a consistent profile of beneficial changes within taxa is lacking [10,11,50,51,52]. The lack of concurrence in these studies is likely a result of the varying methods of husbandry, feed, source and breed of chicks, tissue type and time of sample collection, and overall sequencing and analytical methods. The microbiome of the GIT is temporally dynamic and influenced by intra- and extra-host factors. The primary role of AGPs, and subsequently DFMs, is likely not to induce a defined beneficial microbiome per se, but rather to perpetuate the establishment and maintenance of a beneficial microbial genetic and metabolic profile in the host GIT. As metabolic genes are conserved across genera, the innate metabolic properties of the microbiome appear to be more important for host performance than the specific genera [4] that are present. The new DFM described here increased bacterial diversity, which in turn may establish a larger and more favorable bacterial metabolic profile, which helps the broiler efficiently utilize feed and overcome intestinal dysbiosis. 

In Experiment 2, litter from a commercial farm was used and the broilers were orally given a non-defined challenge to induce intestinal dysbiosis with the goal of replicating potential stressors of a large-scale commercial farm operation. The results support the inclusion of this new DFM in feed at a rate of 0.3 kg/ton as an alternative to an AGP (BMD 11%) to enhance the growth performance of broilers and blunt the negative effects of on-farm microbial stressors. 

The heterogeneity in the *Bacillus* genera, varying spore concentrations, and formulations of DFMs used makes individual comparisons between strains and studies difficult. Luise et al. recently analyzed the results from 131 studies utilizing *Bacillus* spp. DFMs and found “The benefits of *Bacillus* strains on these [growth] parameters [of broilers] showed results comparable to the benefit obtained by the use of antibiotics [5]”. These benefits are received through four primary means: (1) direct effect on pathogenic bacteria, (2) favoring the colonization of the gut by beneficial bacteria, (3) host immunostimulatory effects, and (4) contributions to feed efficiency [53,54,55]. *Bacillus*-based DFMs are a safe and commensurate alternative to AGPs in broilers. 

## 5. Conclusions

In summary, using both a defined (Exp 1, Table 8) and undefined dysbiosis-inducing challenge model (Exp 2, Table 9), chickens fed this new DFM had an observable growth advantage over basal-fed-diets, and the productive growth parameters of the DFM-fed chickens were at least equal to or numerically better than the AGP-fed groups with no observed negative side effects, indicating Zymospore^®^ is a safe and effective AGP substitute for the poultry industry. 

## Figures and Tables

**Table 1:** Colony morphology description of selected *B. subtilis* strains on solid agar medium.

Strain	Form	Texture	Surface	Color	Elevation	Size	Margins
BS-009	Irregular	Rough	Dry	Yellowish white	Flat	0.5–1 cm	Curled
BS-020	Irregular	Rough	Dry	Yellowish white	Flat	0.5–1 cm	Curled
BS-024	Irregular	Rough	Mucoid	Yellowish white	Flat	0.5–1 cm	Curled/Lobate

**Table 2:** Ingredient and nutrient composition of the experimental diets fed to broilers in Experiment 1.

Item	Pre-Starter (1 to 7 d)	Starter (7 to 21 d)	Grower (21 to 35 d)	Finisher (35 to 42 d)
Ingredients, %				
Corn	48.41	50.63	57.05	63.43
Soybean meal	44.03	41.71	35.31	29.96
Soybean oil	3.74	4.35	4.70	4.22
Dicalcium phosphate	1.13	0.83	0.64	0.29
Limestone	1.38	1.23	1.14	0.97
Salt	0.55	0.52	0.50	0.47
DL-Met, 99%	0.35	0.33	0.28	0.24
L-Lys HCl, 78%	0.13	0.13	0.15	0.17
L-Thr, 98.5%	0.05	0.05	0.04	0.03
Choline chloride, 60%	0.03	0.04	0.05	0.07
Vitamin and mineral premix ^1^	0.21	0.19	0.16	0.16
Nutrient and energy composition, % or as shown				
ME, Mcal/kg	2.97	3.05	3.15	3.20
Crude protein	24.16	23.27	20.86	18.92
Ca	1.01	0.88	0.79	0.63
Av. P	0.48	0.42	0.37	0.30
Na	0.23	0.22	0.21	0.20
Cl	0.42	0.41	0.40	0.40
Choline, mg/kg	1600	1600	1500	1500
Lys dig. ^2^	1.31	1.26	1.12	1.01
Met + Cys dig.	0.66	0.64	0.56	0.50
Thr dig.	0.98	0.94	0.84	0.76
Trp dig.	0.86	0.83	0.74	0.67
Arg dig.	0.28	0.27	0.24	0.21
Val dig.	1.53	1.47	1.29	1.15
Ile dig.	1.01	0.97	0.87	0.78
Leu dig.	0.95	0.91	0.80	0.72

^1^ Composition per kilogram of feed: vitamin A, 8000 UI; vitamin D_3_, 2000 UI; vitamin E, 30 UI; vitamin K_3_, 2 mg; thiamine, 2 mg; riboflavin, 6 mg; pyridoxine, 2.5 mg; cyanocobalamin, 0.012 mg; pantothenic acid, 15 mg; niacin, 35 mg; folic acid, 1 mg; biotin, 0.08 mg; iron, 40 mg; zinc, 80 mg; manganese, 80 mg; copper, 10 mg; iodine, 0.7 mg; selenium, 0.3 mg. Ronozyme HiPhos (GT) with 10,000 FYT/g (Novozymes A/S, Bagsvaerd, Denmark). ^2^ Ratios of digestible amino acids to digestible Lys were maintained at TSAA 0.75; Thr 0.65; Val 0.77; Trp 0.17; Arg 1.08; Ile 0.67 [28].

**Table 3:** Ingredient composition and nutrient content of the commercial feed diets used in Experiment 2 on as-is basis.

Item	Pre-Starter (1 to 14 d)	Finisher (15 to 42 d)
Ingredients (%)		
Corn	54.75	57.99
Soybean flour 46%	20.15	0.00
Deactivated soybean	0.00	16.00
Soybean expeller	20.00	18.14
Wheat	0.68	4.79
Grit	1.39	1.06
Salt	0.42	0.40
Mycotoxin binder	0.30	0.30
Dicalcium phosphate	1.16	0.54
Lysine	0.25	0.18
Methionine powder	0.37	0.25
Threonine	0.08	0.03
Choline chloride	0.10	0.08
Trace mineral premix ^1^	0.10	0.10
Vitamin premix ^2^	0.15	0.10
Nutrient and energy composition, % or as shown		
ME, Mcal/kg	2.95	3.10
Crude protein	21	18
Ca	1.02	0.82
Av. P	0.45	0.42
Lys dig.	1.2	1.0
Met dig.	0.48	0.40

^1^ Mineral premix supplied the following per kilogram: manganese, 120 g; zinc, 100 g; iron, 120 g; copper, 10–15 g; iodine, 0.7 g; selenium, 0.4 g; and cobalt, 0.2 g. ^2^ Vitamin premix supplied the following per kilogram: vitamin A, 20,000,000 IU; vitamin D_3_, 6,000,000 IU; vitamin E, 75,000 IU; vitamin K_3_, 9 g; thiamine, 3 g; riboflavin, 8 g; pantothenic acid, 18 g; niacin, 60 g; pyridoxine, 5 g; folic acid, 2 g; biotin, 0.2 g; cyanocobalamin, 16 mg; and ascorbic acid, 200 g.

**Table 4:** Relative enzyme activity (REA) of the three *B. subtilis* strains in Zymospore^®^ (mean ± SD).

Strain	Cellulase	Amylase	Lipases	Proteases
BS-009	3.55 ± 0.34	1.95 ± 0.08	3.16 ± 0.10	2.33 ± 0.09
BS-020	2.02 ± 0.08	1.71 ± 0.13	3.12 ± 0.40	1.96 ± 0.11
BS-024	2.30 ± 0.19	1.38 ± 0.05	2.32 ± 0.19	2.51 ± 0.16

**Table 5:** Temperature resistance of spores of the three *B. subtilis* strains in Zymospore^®^. Data represent the mean ± SD of vegetive cell counts (log_10_ CFU/mL) post-treatment.

Strain	15 °C		37 °C		45 °C	
	2 h	4 h	2 h	4 h	2 h	4 h
BS-009	7.03 ± 0.26	7.13 ± 0.32	7.42 ± 0.10	7.40 ± 0.30	7.20 ± 0.17	6.77 ± 0.68
BS-020	6.97 ± 0.06	7.20 ± 0.35	6.40 ± 0.17	6.30 ± 0.30	7.30 ± 0.0	7.55 ± 0.81
BS-024	7.26 ± 0.24	7.16 ± 0.15	6.95 ± 0.09	6.95 ± 0.09	7.10 ± 0.17	6.93 ± 0.13

**Table 6:** Salinity resistance of spores of the three *B. subtilis* strains in Zymospore^®^. Data represent the mean ± SD of vegetive cell counts (log_10_ CFU/mL) post-treatment.

Strain	NaCl 3.5%		NaCl 6.5%	
	2 h	4 h	2 h	4 h
BS-009	7.14 ± 0.15	7.33 ± 0.35	6.92 ± 0.08	6.77 ± 0.07
BS-020	7.28 ± 0.04	7.15 ± 0.32	7.36 ± 0.10	7.03 ± 0.05
BS-024	7.12 ± 0.21	7.15 ± 0.15	6.96 ± 0.34	6.73 ± 0.15

**Table 7:** Acid resistance of spores of the three *B. subtilis* strains in Zymospore^®^. Data represent the mean ± SD of vegetive cell counts (log_10_ CFU/mL) post-treatment.

Strain	pH2		pH3	
	2 h	4 h	2 h	4 h
BS-009	6.93 ± 0.08	7.33 ± 0.61	7.32 ± 0.28	6.87 ± 0.11
BS-020	7.16 ± 0.28	7.14 ± 0.29	7.01 ± 0.02	6.62 ± 0.54
BS-024	6.95 ± 0.09	6.67 ± 0.58	6.98 ± 0.03	6.95 ± 0.05

**Table 8:** Evaluation of different concentrations of the DFM (0.2 kg/t, 0.3 kg/t, or 0.3 kg/t) on body weight, body weight gain, feed intake, accumulated feed conversion ratio (FCR), and total mortality in broiler chickens given a defined challenge to induce dysbiosis at 42 days of age. Experiment 1.

Item	1 d	7 d	14 d	21 d	28 d	35 d	42 d	ADG ^1^
Body weight (g)								
Negative control	45	200	526	990	1747	2449	3294	77.35
DFM, 0.2 kg/t	45	199	531	1008	1767	2482	3340	78.45
DFM, 0.3 kg/t	45	205	543	1026	1805	2537	3402	79.93
DFM, 0.4 kg/t	45	200	536	1014	1772	2497	3353	78.77
Positive control	45	196	537	1006	1753	2462	3328	78.15
SEM ^2^	0.49	5.72	18.70	26.33	36.02	73.29	77.10	1.834
*p*-value	0.8416	0.1843	0.6004	0.2720	0.0766	0.3002	0.2116	0.2103
Body weight gain (g/d/b)	1–7 d	7–14 d	14–21 d	21–28 d	29–35 d	35–42 d		
Negative control	155	326	554	667	702	845		
DFM, 0.2 kg/t	154	331	568	668	715	858		
DFM, 0.3 kg/t	160	338	573	689	731	865		
DFM, 0.4 kg/t	155	335	568	668	725	856		
Positive control	151	341	559	656	710	865		
SEM	5.66	15.01	16.33	33.50	76.68	57.46		
*p*-value	0.1596	0.4425	0.2720	0.5449	0.9644	0.9702		
Feed intake (g/b)	1–7 d	7–14 d	14–21 d	21–28 d	29–35 d	35–42 d		
Negative control	182	426	738	989	1186	1298		
DFM, 0.2 kg/t	176	424	753	965	1165	1307		
DFM, 0.3 kg/t	182	432	757	965	1193	1300		
DFM, 0.4 kg/t	180	430	758	960	1186	1301		
Positive control	173	433	739	963	1184	1312		
SEM	7.14	15.95	25.12	74.31	142.10	87.72		
*p*-value	0.1240	0.8236	0.4647	0.9574	0.9979	0.9987		
FCR ^3^	1–7 d	7–14 d	14–21 d	21–28 d	29–35 d	35–42 d		
Negative control	1.171	1.310	1.333	1.483	1.696	1.542		
DFM, 0.2 kg/t	1.142	1.282	1.325	1.443	1.637	1.530		
DFM, 0.3 kg/t	1.143	1.279	1.321	1.400	1.631	1.502		
DFM, 0.4 kg/t	1.156	1.283	1.335	1.437	1.640	1.519		
Positive control	1.142	1.271	1.320	1.464	1.664	1.516		
SEM	0.0235	0.0349	0.0237	0.0694	0.1368	0.0929		
*p*-value	0.1742	0.4011	0.7216	0.3314	0.9188	0.9589		
Mortality (%)	1–7 d	7–14 d	14–21 d	21–28 d	29–35 d	35–42 d	Total mortality	
Negative control	0.67	0.67	0.00	0.76	0.72	0.67	3.51	
DFM, 0.2 kg/t	0.00	2.17	0.67	0.00	0.76	0.67	4.29	
DFM, 0.3 kg/t	0.67	2.17	0.67	2.25	0.00	0.67	6.51	
DFM, 0.4 kg/t	0.00	0.67	0.00	0.72	0.76	0.00	2.18	
Positive control	0.67	2.17	0.00	0.00	0.79	0.00	3.70	
SEM	1.26	3.05	1.03	1.59	1.66	1.26	4.73	
*p*-value	0.7359	0.7826	0.5674	0.1272	0.9063	0.7359	0.6174	

Negative control—no antibiotic growth promoter. Positive control—feed supplemented with flavomycin at 25 g/ton. ^1^ ADG = average daily gain. ^2^ SEM—pooled standard error of the mean. ^3^ FCR—feed conversion rate, FI (d 1–42)/BW (d 42).

**Table 9:** Evaluation of Zymospore^®^ on body weight (BW), feed intake (FI), accumulated feed conversion ratio (FCR), and total mortality in broiler chickens given a non-defined challenge. Experiment 2.

	BW g/broiler (d 42)	FI g/broiler (d 1–42)	FCR ^1^ (d 1–42)	Total Mortality (d 1–42)
Basal diet without filtrate (NC−)	2810 ^b^	5302	1.97 ^a^	5/100 (5.00%)
BMD without filtrate (BMD−)	3161 ^a^	5319	1.76 ^b^	3/100 (3.00%)
BMD with filtrate (BMD+)	3055 ^a^	5402	1.82 ^b^	3/100 (3.00%)
DFM with filtrate (DFM+)	3108 ^a^	5456	1.83 ^b^	3/100 (3.00%)
SEM ^2^	110	3891	0.04	
*p*-value	0.0011	0.6784	0.0007	

NC−: Commercial feed formula with no additives and not challenged. BMD−: Commercial feed formula with the addition of BMD and no challenge. BMD+: Commercial feed formula with the addition of BMD and challenged. BMD 11% was included at a rate of 0.5 kg/metric ton in all the feed phases. DFM+: Commercial feed formula with the addition the DFM and challenged. Zymospore^®^ DFM was included at a rate of 0.3 kg/metric ton in all the feed phases and challenged. ^1^ FCR—FI (d 1–42)/BW (d 42). ^2^ SEM—pooled standard error of the mean. ^a,b^ Values within columns with different superscripts differ significantly (*p* < 0.05); *n* = 5 replicates per treatment and *n* = 20 broilers/replicate.

## Data Availability

Not applicable.

## Supplementary Materials

The following supporting information can be downloaded at: https://www.mdpi.com/article/10.3390/ani12111436/s1, Table S1. Growth inhibition of *Salmonella* and *Escherichia coli* isolates by *Bacillus subtilis* on SPA agar plates; Figure S1: Growth inhibition of the select pathogen isolates by Bacillus subtilis isolate BS-009 on Spizizen potato agar (SPA) plate. SE, Salmonella Enteritidis; ST, Salmonella Typhimurium; EC-C, avian pathogenic Escherichia coli (APEC); EC-P, Escherichia coli isolated from a pig; EC-E, enteropathogenic Escherichia coli (EPEC); Figure S2: Order level proportions across individual samples for each treatment group and day of sampling. The height of each color-coded portion of the bar plot represents the percentage of that Order relative to the total number of 16S sequences classified to a taxonomic Order level identified in that sample. Individual replicates (Rn) for each respective treatment group and sampling timepoint are shown. Only Orders with ≥1% proportionality are shown.
