# Supplementary material for: Development and Evaluation of a Commercial Direct-Fed Microbial (Zymospore®) on the Fecal Microbiome and Growth Performance of Broiler Chickens under Experimental Challenge Conditions
Source: Animals (Basel). 2022 Jun 2;12(11):1436. doi: 10.3390/ani12111436 (PMC9179881; doi:10.3390/ani12111436)
Supplement: Supplementary file 1 [file animals-12-01436-s001.zip › animals-1710512-supplementary.pdf]

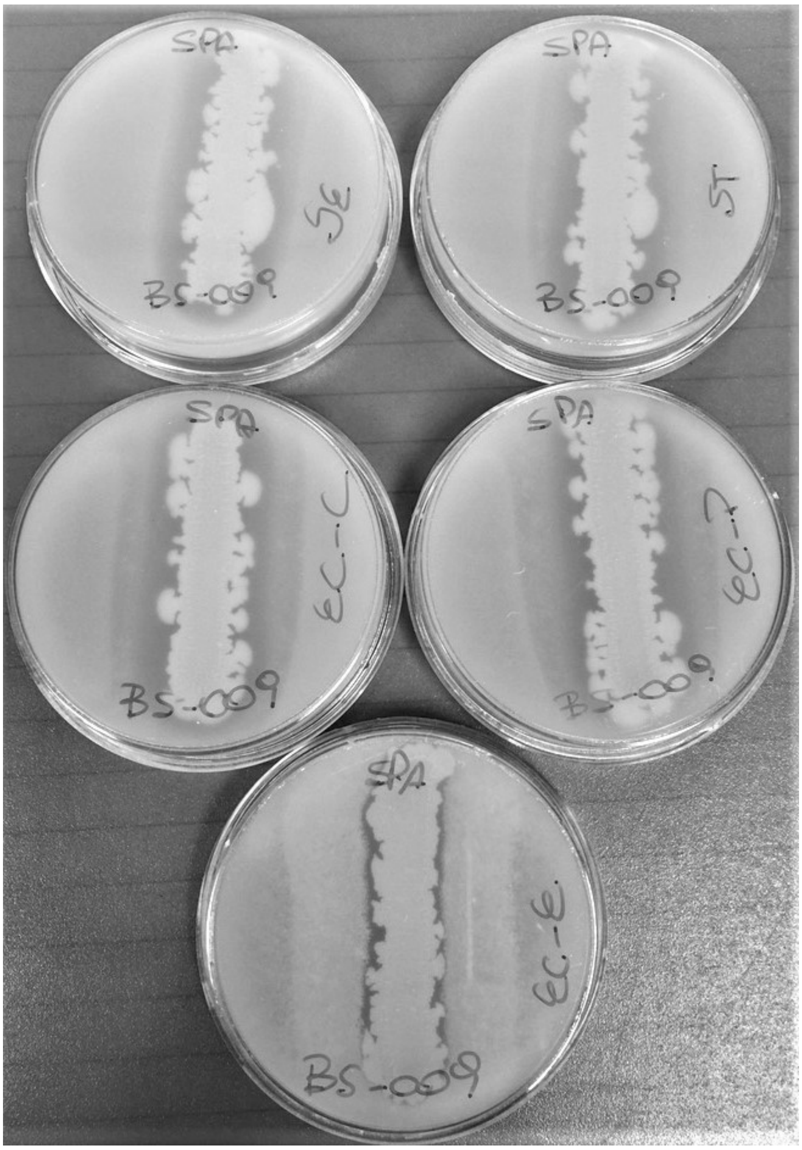

Figure S1. Growth inhibition of the select pathogen isolates by *Bacillus subtilis* isolate BS-009 on Spizizen potato agar (SPA) plate. SE, *Salmonella* Enteritidis; ST, *Salmonella* Typhimurium; EC-C, avian pathogenic *Escherichia coli* (APEC); EC-P, *Escherichia coli* isolated from a pig; EC-E, enteropathogenic *Escherichia coli* (EPEC).

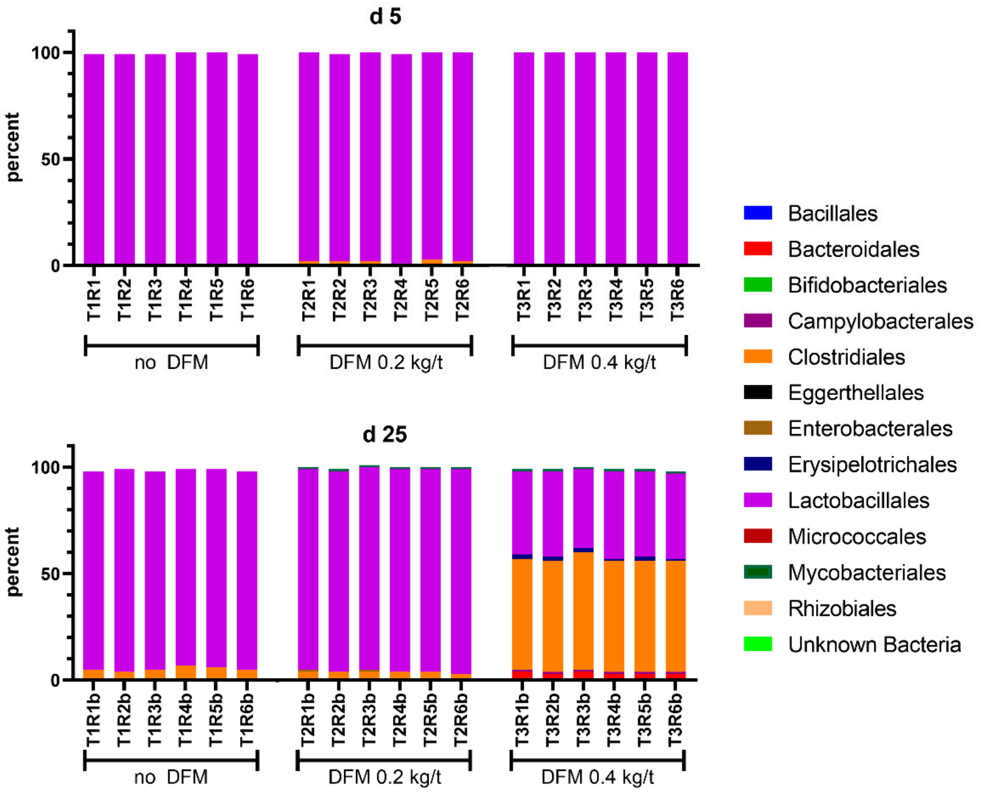

Figure S2. Order level proportions across individual samples for each treatment group and day of sampling. The height of each color-coded portion of the bar plot represents the percentage of that Order relative to the total number of 16S sequences classified to a taxonomic Order level identified in that sample. Individual replicates (R_n) for each respective treatment group and sampling timepoint are shown. Only Orders with ≥ 1% proportionality are shown

Table S1. Growth inhibition of *Salmonella* and *Escherichia coli* isolates by *Bacillus subtilis* on SPA agar plates.

**Isolate #**	**SE**	**ST**	**EC-C**	**EC-P**	**EC-E**
BS-001	++	++	++	++	−
BS-002	++	++	++	++	−
BS-003	++	+	++	++	−
BS-004	+	++	+	++	−
BS-005	++	++	+	+	+
BS-007	++	+	+++	+++	++
BS-008	+	++	++	++	ND
BS-009	+++	+++	+++	+++	+
BS-010	+	+	+	ND	ND
BS-011	+++	+++	++	+++	−
BS-013	++	++	++	+	−
BS-014	++	++	+++	+++	−
BS-020	+++	++	+++	+++	−
BS-023	++	++	+	+	−
BS-024	++	++	++	++	−

SE, *Salmonella* Enteritidis; ST, *Salmonella* Typhimurium; EC-C, avian pathogenic, *Escherichia coli* (APEC); EC-P, *Escherichia coli* isolated from a pig; EC-E, enteropathogenic *Escherichia coli* (EPEC). (−) Bacteria grew to the edge of the *B. subtilis* streak. (+) Narrow halo around *B. subtilis* streak, < 2.0 cm. (++) Halo around *B. subtilis* streak, 2.1—4.5 cm. (+++) Halo around *B. subtilis* streak, > 4.6 cm. (ND) Growth inhibition not determined.
